# Medical and Physical Intelligence

**Published:** 1801-09

**Authors:** 


### MEDICAL AND PHYSICAL INTELLIGENCE.

### [ FOREIGN AND DOMESTIC. ]

### *Van Mons, on the Rhus Radicans, or Toxicodendron.*

(Extracted from a Memoir in the Actes de la Société de Medicine, Chirurgie, et Pharmacie, etablie à Bruxelles, Tome I.)

This Memoir is divided into five sections. 1. Botanical description of *Rhus radicans*, 2. On the poison of *Rhus radicans*, its effects on the human body, and the means of preventing them. 3. On the nature of the deleterious exhalations of *Rhus radicans*. 4. Chemical analysis of the plant, in which a peculiar principle seems to exist; on the properties and nature of this principle. 5. On the effects of *Rhus radicans* as a remedy, its specific virtues in palsies and herpetic diseases.

The ancients had but a very imperfect knowledge of the *Rhus radicans*, or *Toxicodendron*, both which species of LINNÆUS ought to be united under one; which opinion Cit. VAN MONS is inclined to adopt on the authority of TOURNEFORT; and he adds a full botanical description of the plant, with a figure of it.

It has been generally asserted, that the venomous part of this plant is lodged in the milky juice which it yields; more accurate observations, however, have shown that the deleterious effects of the *Rhus radicans* originate in a gaseous substance, which is disengaged from the living plant, whereas the dry and withered leaves are quite deprived of it. The effects produced by this plant in the human body differ according to the constitution of the persons that expose themselves to its action; and also according to the circumstances under which it acts on the human body. For it has been remarked, that this gas is almost without any effect during the time when the plant is exposed to the direct rays of the sun; but it becomes very efficacious when it is exhaled during night, in the shade, or on a rainy day; from which phenomenon Cit. Van Mons is induced to think, that the deleterious exhalation is a gasiform juice, imperfectly elaborated by the plant; an idea which seems to be confirmed by the observation that the gas exhaled in sunlight appeared to be nothing but pure oxygen gas, whereas the gas exhaled during night, consisted of hydrogen and carbon; from which circumstance he concludes that the basis of the pernicious gas is retained in the plant, and changed into the substance of the plant by the action of the sun. The effects which are produced by the poison of *Rhus radicans*, consist in a tumour and swelling of the head, a burning sensation or itching in the skin, and in blisters that rise on the skin. The common effect of the poisonous exhalations in in our climate, is a burning fenfation at the fore-arm and neck, which however difappears in a fhort time. By the chemical analyfis of the plant, the following refults have been obtained: 1. The prevailing fubftance is a peculiar principle, an hydro carbon, which is extremely combuftible, and is contained in the ftalk as well as in the leaves. 2. A great deal of tanin or tanning principle is likewife to be found in the plant. 3. Gallic acid, and but a little colouring matter, notwithftanding the dark green of the leaves. 4. No refin, but fome gum. From this account, it appears with what little reafon the effects of that plant have been formerly attributed to the juice, the nature of which feemed to be unknown.

Cit. DUFRESNOY was the firft who difcovered the great efficacy of the Rhus radicans in palfies and herpetic difeafes, it being never employed before him as a medicine. His obfervations have been confirmed by numerous experiments made by practitioners of different countries.

The beft mode of adminiftering the *Rhus radicans* is undoubtedly in form of an extract, which, as may appear from the above ftatements, is entirely deprived of its noxious particles. The firft time, however, it was employed with much precaution, and the dofes no higher than three pills every day, each confifting of five grains; but it was afterwards obferved that it might be given without injury in the dofes of one ounce a day. DUFRESNOY combines the internal ufe of the plant with the external application of the oil of this plant, with which the affected parts are to be rubbed three times a day. It is made by a hot infufion of the ftalks of Rhus radicans and the hyofciamus (in the proportion of 2 : 6.) in the oil of olives, which is fuffered to ftand fifteen days. The extract may be prepared in five different ways; from the frefh leaves by expreffion, from the dry leaves, from the juice, &c. all which preparations are accurately defcribed in the Memoir. DUFRESNOY gives the preference to the extract made of the frefh leaves, but VAN MONS to that of the dry leaves. This excellent Memoir terminates with the analyfis of a pamphlet of Prof. WURZER on the *Rhus radicans*, in which, feventeen cafes of palfies of the inferior extremities, and of hemiplegies are related, which were cured by the Rhus radicans, to which he adds a cafe of a paralyfis hepatitis, and of a convulfive diforder, in which that plant proved fuccefsful. We conclude this extract with the wifh that this new remedy may ftand the teft of experience and impartial obfervation.

---

### *Obfervations on the good Effects of Acetic Ether, applied in Frictions against Rheumatic Paroxysms and the Gout by* Cit. DESPARANGES.

The effects of acetic ether againft rheumatifm have been confirmed by repeated obfervations, efpecially by Cit. MARTIN, whofe remarks we communicated in a former number of this Journal.

We now beg leave to add two obfervations of Cit. Desparanges, tending to prove the fuccefsful application of that remedy in a cafe of gout, and in another of rheumatifm.

Obs. I.—Cit. Blondeau, fifty years of age, of a bilious temperament, had been afflicted three years with a violent lumbago, that attacked him particularly in the fpring and autumn. In the beginning of May, 1799, he was feized with the moft violent pains, for which feveral remedies were employed in vain for three days. At laft he had recourfe to the acetic ether, with which the frictions were made in the dofe of three drachms. In a fhort time his fufferings confiderably abated; at the end of five frictions the patient was again able to walk, and felt but little fenfation of pain, and fince that time he has never fuffered a new paroxyfm.

Obs. II.—Mrs. Chartran, aged 51, of a fpare habit, and dark complexion, had been fuffering for two years violent ifchiadic pains, which increafed to fuch a degree that they were almoft infupportable. Cit. Desparanges was called to attend her; he found an inflammation and fwelling at the articulation of the thigh with the trunk, the reft of the thigh being neither fwelled nor inflamed, but only painful. The patient was without fever. Under thefe circumftances, Cit. Desparanges did not think it proper to delay the application of acetic ether in frictions. He began with two drachms, which however had no effect the two firft times, but the dofe being increafed at the third time to half an ounce in the morning and evening, the pains became lefs violent, and the patient had a copious topical perfpiration. This treatment being continued fix days, a general perfpiration enfued, after which the patient could rife a little; on the tenth the pains difappeared almoft entirely, and on the twelfth the cure was complete.

---

### Cit. Chaussier's *Method of preserving Anatomical Preparations.*

Citizen Chaussier has communicated to the Society of Medicine at Paris, a new method of preferving animal fubftances. After having enumerated the different methods employed for that purpofe, he points out their defects and infufficiency. For preparing parchment, the fkins are macerated in water, difengaging from them the unctuous particles, and diffolving likewife a part of the mucilage, fo that nothing remains but the fibrous part. For tanning, the hides are put into lime-water, fulphuric acid, or into a folution of alkali, and afterwards expofed to the action of the *tanin* or tanning principle. For preferving anatomical praparations, they are generally put into alkohol, which dries them up and entirely changes their proper form; and though they are in this way preferved from putrefaction, yet they do not remain untouched by the infects. The carbonate of foda, and the fulphat of iron, which are alfo ufed for keeping off putrefaction from animal fubftances, render them foluble in water by combining themfelves with the unctuous particles, and forming a foap with them, whereby the fize and form form of the preparations are confiderably altered. In order to avoid all thefe inconveniencies, Cit. Chaussier fuffers the parts intended for prefervation to be macerated during a longer or fhorter time, from three to eight or ten days, according to their refpective fize, in a folution of oxygenated muriat of mercury in diftilled water. This liquor being always kept in a perfect ftate of faturation, by adding from time to time frefh muriat of mercury for that which is decompofed, imparts a great folidity to the parts impregnated with it, by giving confiftency to the gelatinous parts without changing their fize and form, and when expofed to the air for fome time, they are fecure from corruption and infects. It is through the medium of this mode of preparation, that Cit. Chaussier has made feveral interefting obfervations on the ftructure of the brain, and particularly of the fpinal marrow, for he difcovered that this part, after being deprived of its *pia mater*, is compofed of fix very diftinct bundles; farther, that all the nerves which arife from this part of the brain are by no means fimple productions of its fibres, but that they are inferted in it like hairs, by means of bulbs which adhere to the medulla by feveral fmall roots; and when thefe nerves are pulled out, a double row of fmall regular holes will appear to the eye, into which the bulbs are implanted. A portion of brain, prefented to the Society by the inventor of this method, had the folidity of wood, without the leaft change in its natural fize and form; another brain and fpinal marrow prepared in the fame way, very diftinctly fhowed the holes into which the nerves were ingrafted. The celebrated Ruysh made alfo ufe of a liquor and of injections to preferve his excellent anatomical preparations, by means of which he had fucceeded in preferving the body of his own daughter, in the colour of life and frefhnefs of youth. This liquor, which he always kept as a myftery, feems to be the fame with that of Cit. Chaussier, or at leaft fomething analogous. According to Ruysh, it likewife made the gelatina folid, and by degrees as hard as wood; the albuminous matter coagulated by it, and the cryftalline lens put into it, became opacous and white; the brain obtained in it a cafeous and folid confiftency. For colouring the injections, it is advifable to take madder or cinnabar, but never to make them of wax, or any other unctuous matter, but of a mucilaginous folution, as the folution of *ichthyocolla*, or ifinglafs. After having injected the parts, they ought to be put into the above folution of oxygenated muriat of mercury, where the matter for injecting concretes, and becomes folid. For preferving whole bodies, it is neceffary to make openings into the great cavities, head, cheft, and belly, large enough for the liquor to penetrate into them, as without this precaution the inteftines will not be fecured from corruption. Ruysh himfelf always made fuch incifions for the above purpofe.

---

Prof. Himly, of Brunfwick, has prefented a Memoir to the Royal Society of Sciences of Gottingen, in which he communicates fome interefting remarks on the paralyfis of the pupil, occafioned by the topical application of *extractum hyosciami* on the eye, similar to that which has been observed from the use of *Belladonna*, (Medical and Physical Journal, vol. iii. p. 367,) and the *aqua Laurocerasi*. The first observation was made in a very obstinate case of weakness of sight, where a known remedy, of *one scruple* of extractum hyosciami dissolved in *one* ounce of distilled water, had been applied on the margins of the eye-lids, after which, a most surprising dilatation of the pupil ensued, that lasted for a considerable time. This experiment has been repeated by Prof. HIMLY on more than twenty persons, by letting fall a few drops of this solution into the eye, in which it ought to be kept for a short time by bending the head backward. It never failed to produce the intended effect, if the pupil itself had any power of motion left. The great dilatation of the pupil appears one or two hours after the application of the extract, and generally lasts for five or six hours, without in the least affecting the retina. The water distilled from the fresh herb of hyosciamus had little or no effect, and likewise the *emplastrum de hyosciamo*, or *de belladonna*. The author adds a few remarks on the use that is likely to result from the above experiment, which are the more interesting as his own experience has furnished him with observations, where it proved of some service. According to them, it may be employed as a diagnostic expedient in the cataract, in order to discover whether the capsule adheres to the iris or not, whether the red spots, which in some cases appear before the eyes of such patients, originate from a vitium retinæ, or from a peculiar red coloured obscuration of the crystalline lens. It may also serve as a palliative remedy in the cataract, when the crystalline lens is only darkened in some places. The author has accurately determined the cases in which that expedient seems to prove advantageous; and he intends treating this subject more copiously in a future publication.

---

Mr. KARSTEN, of Berlin, has repeated the experiments of BOUILLON LA GRANGE on the analysis of cork, and on the peculiar acid that is contained in it, (Medical and Physical Journal, vol. iii. p. 89,) whose principal character consists in dissolving with great difficulty in water, on which account it can be only obtained in form of a powder. The acid, however, which Mr. KARSTEN extracted from cork, shoots into crystals that are freely soluble in water. From the other characters which this acid shewed, it appeared to be nothing but oxalic acid. Mr. KARSTEN, however, is inclined to ascribe this difference of results to the difference of cork that was respectively used for the analysis.

---

Mr. NŒF, surgeon of Wermgerode, relates two cases of carious ulcers, in which he employed the juice of *plantago angustifolia* with the greatest success, the use of which remedy is also much recommended by Prof. ARNEMAN, who found it likewise effectual in his own practice.

---

Cit.

*Cit.* COQUART's *Observations on some Effects of Lightning and Electricity in Gonorrhœa.*

OBSERVATION I.—Towards the end of July, 1800, Cit. N. aged forty, of a very irritable conftitution, had a gonorrhœa during fifteen days, attended in the beginning with inflammatory fymptoms. The patient, however, was fo far recovered by a proper treatment, that the pains, as well as the running, confiderably abated. One night when he was fleeping on his bed, almoft naked, and with the windows open, he was fuddenly awakened by the noife of thunder; he leaped from his bed, and having walked in the chamber for fome minutes, he went to bed again. He, however, immediately felt fome pains in the perinæum, and had a reftlefs night: The next morning he had a complete fuppreffion of urine, and all his attempts to make water produced nothing but a few drops of blood; the running had likewife ceafed. The patient was immediately ordered to be put into a bath, and he then made a little water mixed with blood. About mid-day another bath was ufed, after which the urine proceeded in fmall ftreams, and bloody. When he had taken a third bath in the evening, no more blood came off with the urine; and by a ftrict regimen, the natural flow of the urine was re-eftablifhed. The patient went afterwards into the country, and having difcontinued the baths, the gonorrhœa fpontaneoufly returned; the matter was milky, and not copious; the pains in the perinæum were flight, and only felt at the time of erection. Some time after, the running, and all the other fymptoms of the gonorrhœa difappeared entirely. This obfervation naturally leads to the queftion, whether the fuppreffion of urine is to be attributed to the furprife of the patient, by being fo fuddenly awakened by the noife of thunder, or to an electric action? However, this effect of electricity is not quite fo much proved as in the following obfervation.

OBSERVATION II.—An Englifhman, who had for the fpace of two years, a running from the urethra, againft which he had confulted the beft practitioners in France and England, without the leaft fuccefs, applied at laft to Citizen ROUELLE, phyfician at Loeven, of great reputation. This gentleman having again tried all poffible internal and external remedies to no purpofe, had recourfe to electricity, with which he ufed to amufe himfelf. Having, therefore, introduced into the urinary canal of the patient an iron thread, he drew out of it only one electric fpark, whereupon the patient inftantly felt a vehement pain in the perinæum; the gonorrhœa, however, completly difappeared, and the patient was perfectly cured.

---

A memoir has lately been addreffed to the Medical Society at Paris, containing a very fingular cafe of mental derangement. The fubject is a man, fixty-two years of age, who, in confequence of an hemiplegia, with which he was twice attacked, and of which he was cured by a proper treatment, had loft his memory entirely, fo as not to recollect his own name, nor that of his his wife and children; and only retained the expressions *oui*, yes; *non*, no; *beaucoup*, much; *très bien*, very well; *au charme*, charmingly; *point de tout*, not at all; *c'est vrai*, it is true; *c'est juste*, it is right; *à merveille*, &c. The physician who consults the society, desires to know, Whether there exists a physical or moral treatment, by which this patient may recover his memory? The observation being communicated to the *Societé Philomatique*, the Citizens HALLE and MOREAU have been appointed to give an account of this singular case.

---

Citizen PENON has presented three memoirs to the National Institute, containing some observations on the uterus, in which he particularly examines the successive dilatations of that organ, and of its *cervix*, during the periods of pregnancy, especially in the eighth month. The theory, however, which he attempts to establish, according to his anatomical views, does not at all seem to agree with the principles of modern and sound physiology, which is, for the honour of our times, raised on the ruins of the pure mechanic system. In order to enable our readers to judge of what we have asserted, we shall add here, a fragment of the extract of the above memoirs. Cit. Penon is of opinion, that the aqueous particles, and even the urine, dilute the uterus, by acting on it as emollients; and he thinks it is, for this reason, that Nature has placed the urinary bladder and the uterus so near to one another. He likewise thinks, that the membranous bursa, furnished to the uterus by the peritonæum, does not penetrate between this and the bladder, the last organ being intended to contribute to the dilatation of the uterus during the time of pregnancy, for which purpose it ought to be contiguous with the uterus, into which it lets pass, through its pores, a part of its liquor, for mollifying it, which, he imagines, will be greatly supported by injecting water into the uterus. Having observed that acids impart a very great elasticity to the substance of the uterus, he is led to think, that the milk, which is deposited after delivery into the cellulæ of these organs, may contribute to the contraction of the uterus by becoming sour. Such are the gross ideas of the author, which we have merely communicated for the purpose of showing how unacquainted some people are with the improvements of our times, as the above theory would scarcely have done honour to the darker ages of science. It is still more to be wondered, that these results of a mistaken imagination, were admitted before so respectable a body as the National Institute of France, which is occupied in spreading true knowledge and enlightened principles.

---

Cit. LASSUS imagines, that the hernia umbilicalis originates, in many cases, from the too great size of the liver, which sometimes increases in an extraordinary manner from a particular disposition. This morbid conformation terminates, in most cases, fatally.

---

Amongst

Amongst the interesting contents of the *Journal de Physique*, the memoir of Cit. Cuvier has particularly engaged our attention. The author remarks, that notwithstanding the most assiduous researches, we have not yet succeeded in uniting the different phenomena of voice under a certain theory, a circumstance which, he thinks, is partly owing to anatomists not being sufficiently acquainted with the nature of musical instruments, partly to their having merely confined their inquiries to the human voice, the most complicated of all, and least similar to the instruments of music which we know. In order, therefore, to proceed from the simple to the compound, and by that means to find out, by induction, new results, Cit. Cuvier has thought it proper to study the organ of voice in birds, and afterwards to make an attempt at explaining the human voice. His memoir may be reduced to the following articles:

1. *On the researches of other anatomists concerning the voice of birds;* where he gives an account of the works of Casserius, Perrault, Herissant, Vicq. d'Azyr, Bloch, and of his own ideas, published in one of the numbers of the *Magasin Encyclopedique.*

2. *On the place where the voice of birds is formed:* In which article he states, that for producing sounds by means of a wind instrument of music, the intervention of an elastic body is necessary, that is put into vibration, and that the tube only modifies the sound produced at its mouth by the sonorous body which is put into vibration. In man, and other animals of the mammiferous tribe, the mouth and nasal cavities are the only parts that may be compared with the tube of an instrument, the sound forming itself at the end, and in the upper part of the trachea. The construction of the vocal instruments in birds differs in a great measure from this, because their trachea, or wind-pipe, modifies the sound which is formed at its lower extremity, at which place a true glottis exists. This observation is confirmed by three experiments, according to which, a black-bird, a magpye, and a duck, continued to cry, notwithstanding their throats being cut through, and the duck even gave sounds after the complete amputation of the neck.

3. *General ideas on the means by which the birds vary the sound.* They consist in the variation of the glottis in the *larynx inferior*, and of the wind-pipe, and in the faculty of contracting and dilatating the superior glottis.

4. *On the larynx inferior.*

5. *On the wind-pipe.*

6. *On the larynx superior.*

These articles are devoted to more detailed researches of comparative anatomy, and we are hence entitled to conclude, that the organ of voice in birds is a true instrument of wind music, of the class of trumpets, and particularly of the sackbuts, (tombon). Cit. Cuvier announces, at the end of this memoir, that he intends to apply his doctrine for explaining the organs of voice in man, and the mammiferous animals.

Cit.

Cit. Demanet is inclined to afcribe, in a great meafure, the caufe of convulfions that affect women during the period of pregnancy and delivery to an *anafarca.* The moft refpectable accoucheurs have only adopted three caufes of convulfions in pregnant women; lofs of blood, its too great abundance, and the pains of parturition. Cit. Demanet, however, relates fix cafes, in which he pretends to have obferved the above caufe; it muft, however, be allowed, that it is difficult to conceive in what manner the convulfions can be occafioned by this fymptom.

---

Mr. Mursinna, of Berlin, has made fome remarks refpecting the cure of tetanus, or locked jaw, a difeafe, the aitiology of which is by no means afcertained. According to the experience of this great furgeon, *opium* ftill remains the moft efficacious and powerful remedy againft that dreadful affection. It ought to be given at the firft appearance of the difeafe, in fmall dofes, and repeatedly adminiftered at fhort intervals. A bath of warm water with a large quantity of foap added to it, greatly advances the cure; and it has been obferved that the patients receive in the bath the power of opening the mouth, and of fwallowing. Opium, however, ought to be given in a liquid form; on which account, according to Mr. Murfinna's experience, the laudanum Sydenhami is moft preferable, as the patient can take it in fufficiently large dofes without perceiving any naufea. There is nothing to fear from too great a dofe of opium, becaufe it muft be given in increafed dofes, till the difeafe begins to abate; and at this period only, the dofes may be leffened. In combination with the internal ufe of opium, blifters, frictions with quickfilver, and opium, may be applied, by which the effect of that remedy is greatly fupported. Mr. Murfinna employed in two cafes, the method of cure propofed by Dr. Stutz, but without fuccefs. The firft was a trifmus and tetanus, which enfued a caftration, eight days after the operation had been performed. The patient died in three days from the beginning of the difeafe. In another cafe the trifmus fupervened twelve days after the extirpation of a fcirrhus, and though the fpafm feemed at firft to abate, and even to difappear by that method of cure, yet it came on again unexpectedly, and put an end to the life of the patient thirty hours after its firft appearance.

---

It is with pleafure we obferve the rapid progrefs of the Vaccine Inoculation on the continent. There is hardly any confiderable place in Germany where it is not practifed, and there exifts a general emulation among phyficians, particularly of that country, to propagate this great difcovery, by which the laft century feems, as it were, to compenfate the bloodfhed and the lofs that the prefent unhappy war has inflicted on Europe, as it offers to us the confoling hope of extirpating the Small-pox, that fcourge of mankind, kind, which for so many centuries has afflicted humanity in its most tender parts. It is particularly with this great view, that several physicians, among whom we find the venerable name of Hufeland, have thought it worthy their attention to take measures, in order to spread the inoculation of the Cow-pox. The physicians of Geneva have published the following advertisement, which, in that important moment when the children are christened, is delivered by the clergyman to the godfathers, after the baptism is over, in order to make the people more inclined for the Cow-pox, by presenting to them the Vaccine Inoculation as a matter of conscience :—"The child that is just christened is, amongst different other dangers, exposed to become a sacrifice to the Small-pox, a disease that, since the eighth century, has spread itself through Europe in such a manner, that it is morally impossible to preserve the child from it, except by inoculation. Luckily, and by a particular favor of Providence, a discovery has been lately made, confirmed by the test of experience, which is both harmless and secure, and may be employed in all seasons, in weak, tender, and new-born children; a remedy that is never attended with any bad symptom, but of the mildest nature, viz. the Vaccine Disease, an affection that has hitherto proved slight, and very mild, and affords the inestimable advantage of not being contagious, so that it may be inoculated in a house without any fear of danger to the neighbours, though it, at the same time, averts for ever the misfortune of receiving the Small-pox. If you, Parents! wish to defend this child from that danger, we conjure you to get it immediately inoculated with the Cow-pox! Hasten to preserve it from that dangerous disease, which daily fills your hearts with desolation, and by which your children risk every moment to be infected; do not even deliver them to the nurses, before you have rescued them from this danger. The experience and observation of the physicians and surgeons of this place, who know themselves the feelings of a father, and who have inoculated their own children, ought to encourage you to follow their example. In the name of humanity, by all that is dear to you, we request you to follow their example; if not, we justify ourselves, when once you shed tears for the loss of this child: You have not been willing to avail yourselves of this remedy that was offered to you, simple and easy as it is; you have refused it; you have unreasonably doubted to apply it in time. We, the subscribed physicians and surgeons, have made it our duty to inoculate every child that has been brought to us, without the least recompence. Our fellow citizens know, that we have hitherto accepted no reward from any person whose circumstances did not allow it."

Signed, *Vieusseux*, *Odier*, *Vignier*, *Monget*, *Veillard*, *Coindet*, *De la Rive*, *Peschier*, physicians; *Jurine*, *Fine*, *Maunoir*, surgeons.

---

The celebrated French mineralogist, Hauy, is about to publish a work on mineralogy, consisting of three volumes, in large octavo. From the abilities of this literatist, we have reason to expect, pect, that it will become one of the most elaborate and instructive works we possess on that branch of natural science.

---

A third volume of MEDICINA NAUTICA is nearly ready for publication, which, for original matter and more careful selection, will surpass the preceding volumes. The medical department, as well as others in the navy, acquired fresh activity from the flag of Lord St. Vincent appearing in the Channel; new facts and observations have been recorded to more advantage; and as the present volume is likely to bring down the occurrences of the fleet to the conclusion of the war, the whole will exhibit a wider field of medical practice in the navy than has ever been reaped by others.—Communications for this work may be addressed to the author, *post paid.*

---

To the EDITORS of the MEDICAL and PHYSICAL JOURNAL.

